# Diagnostic optical coherence tomography findings in arteritic posterior ischemic optic neuropathy: A case report

**DOI:** 10.1016/j.ajoc.2026.102691

**Published:** 2026-10-14

**Authors:** Andreas Worm Bendtsen, Michael Stormly Hansen, Bjarke Askaa, Steffen Hamann

**Affiliations:** a Department of Ophthalmology, Copenhagen University Hospital - Rigshospitalet, Denmark; b Department of Clinical Medicine, University of Copenhagen, Copenhagen, Denmark; c Department of Rheumatology and Spine Diseases, Bispebjerg - Frederiksberg Hospital, Denmark

**Keywords:** Paracentral acute middle maculopathy, OCT, Posterior ischemic optic neuropathy, Giant cell arteritis

## Abstract

**Purpose:**

To describe the finding of paracentral acute middle maculopathy (PAMM) in a patient with posterior ischemic optic neuropathy (PION) leading to the diagnosis of occult giant cell arteritis (GCA).

**Case report:**

A 67-year-old man presented with sudden monocular vision loss. Ophthalmic examination revealed profound vision loss with an ipsilateral relative afferent pupillary defect, a cotton wool spot and PAMM on optical coherence tomography (OCT), while the optic disc appeared normal. Fluorescein and indocyanine green angiography revealed a perfusion deficit in the nasal choroid. There were no systemic symptoms and acute phase reactants were normal. Temporal artery biopsy was negative, while temporal artery ultrasound revealed definite signs of GCA. Accordingly, the final diagnosis of arteritic PION was made. At 9-month follow-up the optic disc was atrophic, and retinal ischemic perivascular lesions (RIPLs) had developed at the previous locations of PAMM.

**Conclusion:**

PAMM on macular OCT may occasionally be the only indication of GCA in patients with sudden vision loss. Concurrent findings of PAMM and ischemic optic neuropathy in patients aged 50 and above should prompt urgent investigation for GCA and consideration of initiating treatment with high-dose corticosteroids, even without classic diagnostic features.

## Highlights

• Macular OCT should be routinely performed in sudden vision loss.
• PAMM may be found concurrently with PION.
• PAMM may be the only indicator of underlying GCA.
• A multimodal diagnostic approach is recommended when GCA suspicion is high.

## 1 Introduction

When assessing sudden onset visual loss in elderly patients, underlying giant cell arteritis (GCA) is an essential diagnosis to consider. GCA is associated with various ocular ischemic disorders, most notably arteritic ischemic optic neuropathy (A-ION).^1^ The anterior variant (A-AION) of A-ION is far more common than the posterior variant (A-PION), which constitutes only around 8-10% of A-ION cases.^1^, ^2^ In A-AION, optic disc edema is always present and on optical coherence tomography (OCT), paracentral acute middle maculopathy (PAMM) has been suggested as a specific biomarker of underlying GCA when differentiating from nonarteritic AION (NA-AION).^3^, ^4^

As there is no optic disc edema in A-PION, diagnosis is difficult and usually only established retrospectively, once optic atrophy has developed. However, in A-ION, time is of the essence. High dose corticosteroid therapy should be initiated immediately upon suspicion of GCA to reduce the risk of bilateral ocular involvement.^1^ It is therefore of utmost importance to find early clinical signs of ocular ischemia, when confronted with a putative case of A-PION.

In around 20% of GCA-related ocular ischemic events, there are no accompanying systemic symptoms at presentation, such as headache, jaw claudication, and scalp tenderness. This has been termed occult GCA.^5^ In occult GCA, blood samples may reveal elevated acute phase reactants including C-reactive protein (CRP) and erythrocyte sedimentation rate (ESR). In around 8% of GCA cases, however, CRP is within normal limits, which may be particularly associated with the ocular ischemic phenotype and occult GCA.^5^, ^6^

In this report, we describe a rare case of A-PION without accompanying GCA symptoms or elevated acute phase reactants, where the findings of PAMM on macular OCT led to the subsequent diagnosis of GCA.

## 2 Case report

A 67-year-old man presented with sudden onset vision loss in the right eye. The visual loss initially involved only the inferior visual field but progressed to the entire visual field within 2 days of onset. Systemically, the patient reported no accompanying symptoms including absence of any headache, scalp tenderness, jaw or tongue claudication, proximal muscle pain, fatigue, loss of appetite, weight loss, fever or any neurological deficits. Prior to the event, the patient had no history of eye disease, while history of systemic disease included mild chronic obstructive pulmonary disease (COPD) and HLA-B27 positive ankylosing spondylitis.

Upon examination, visual acuity was restricted to hand motion in the right eye and was 20/20 in the left eye. The patient was emmetropic and intraocular pressure was 12-13 mmHg in both eyes. A pronounced relative afferent pupillary defect (RAPD) was present in the right eye. Automated perimetry (Octopus, Haag-Streit) revealed complete visual field loss in the right eye with a mean deviation of −26.2 dB and a normal visual field in the left. Ophthalmoscopic examination of the affected right eye revealed a cotton wool spot (CWS) superotemporal to the optic disc and slight narrowing of the arterioles, but was otherwise unremarkable. Optic discs were without edema in both eyes. Palpation of the temporal arteries on both sides was normal. Blood samples revealed mild dyslipidemia but were otherwise unremarkable, including normal hemoglobin (9.9 mmol/L), leukocytes (6.8 x 10^9^/L), CRP (3.1 mg/L), and ESR (2 mm/hour).

OCT of the macula at presentation (OCT Triton, Topcon Healthcare) revealed multiple, diffusely spread hyperreflective lesions of the inner nuclear layer throughout the macula of the right eye, consistent with the definition of PAMM.^7^ The CWS was distinguished from PAMM on OCT by its location in the retinal nerve fiber layer, as opposed to the inner nuclear layer (Fig. 1B). No such lesions were seen in the unaffected eye. OCT of the right optic nerve head was unremarkable except for a slight increase in temporal and superotemporal peripapillary retinal nerve fiber layer (pRNFL) thickness co-localized with the CWS. Global pRNFL thickness was 121 μm, compared with 110 μm in the left eye. Fluorescein and indocyanine green (ICG) angiographies were performed, revealing a significant delay in perfusion of the nasal choroid of 30-40 seconds compared to the temporal choroid. This corresponded to the perfusion area of the medial short posterior ciliary artery/arteries. There were no indications of retinal perfusion deficits or central retinal venous occlusion. Fig. 1A–E shows fundus, OCT, and ICG angiography findings at presentation.

**Fig. 1:**
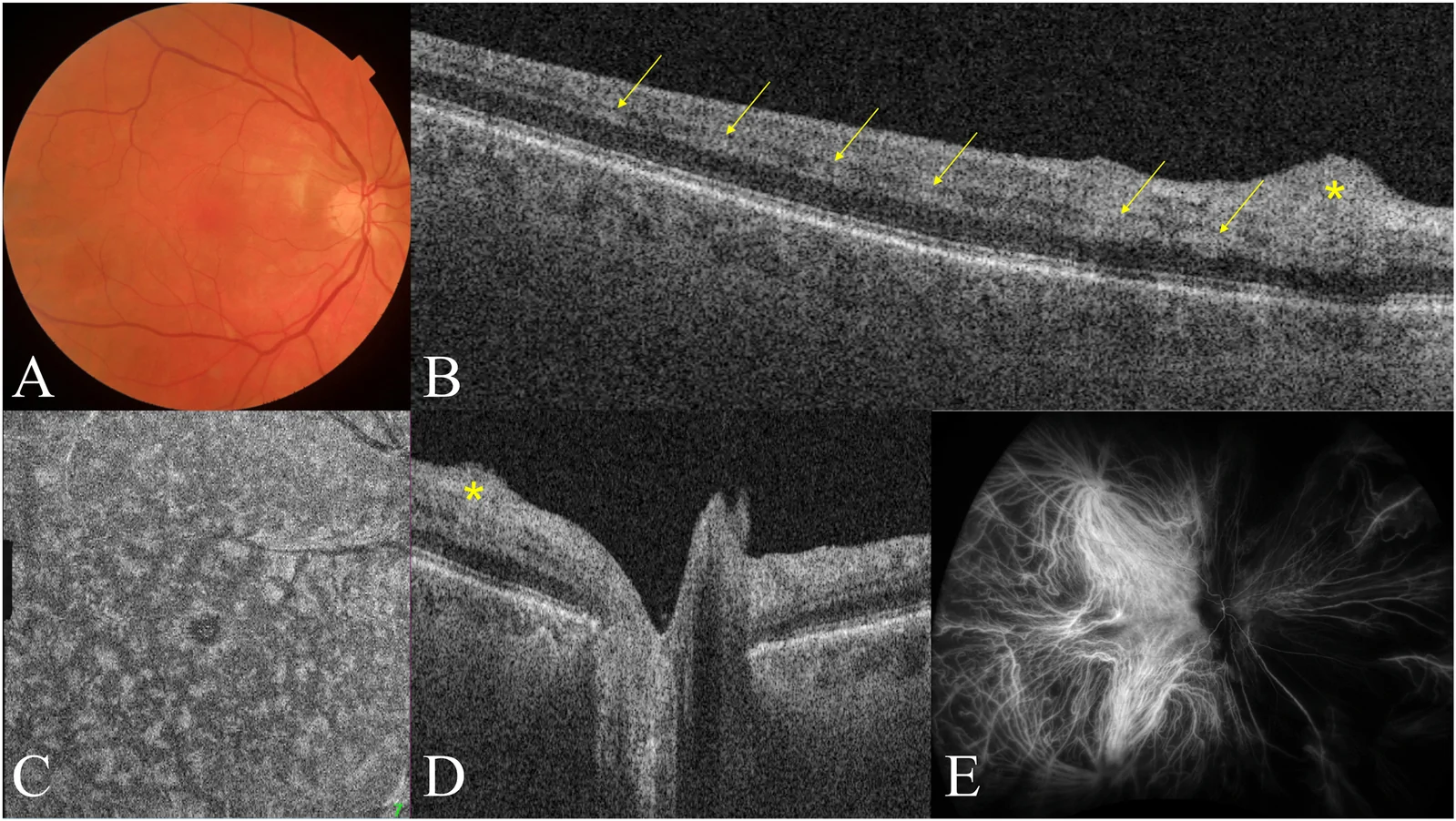
A-E Imaging findings in the affected right eye at presentation. A: Fundus photograph with a cotton wool spot (CWS), slight narrowing of the arterioles, and no optic disc edema. B: Macular optical coherence tomography (OCT) showing paracentral acute middle maculopathy (PAMM) (arrows) and a CWS (asterisk). C: En-face view of macular OCT at the level of the inner nuclear layer showing PAMM in a perivenular pattern. D: OCT through the center of the optic nerve head without optic disc edema. E: Indocyanine green angiography 31 seconds after injection revealing a nasal choroidal perfusion deficit. (For interpretation of the references to color in this figure legend, the reader is referred to the Web version of this article.)

These findings indicated that vision loss resulted from an acute ocular vascular event, and due to the presence of both retinal ischemia on OCT and choroidal perfusion deficits on angiography, clinical suspicion of GCA was raised and high-dose corticosteroid therapy initiated. Owing to the complete absence of other GCA symptoms and normal acute phase reactants, however, the patient was admitted to the emergency neurological department to rule out differential diagnoses. Apart from the monocular visual deficit, neurological examination was normal. Magnetic resonance imaging (MRI) without contrast (stroke protocol) was without signs of cerebral infarction or other acute pathology. However, dual antiplatelet therapy and lipid-lowering therapy was initiated given the low but non-negligible suspicion of a thrombo-embolic event. Carotid duplex ultrasound and 24-h Holter monitoring were without indications of a thrombo-embolic source.

Ensuing biopsy of the right temporal artery (performed 5 days after treatment initiation with a sample size of 25 mm) was without any pathological changes. Temporal artery ultrasound performed 6 days after treatment initiation demonstrated bilateral thickening of the intima-media complex with positive halo sign in the superficial temporal artery branches (Fig. 2A–B). These findings were consistent with active arteritis and the diagnosis of GCA in accordance with local guidelines informed by EULAR recommendations.^8^

**Fig. 2:**
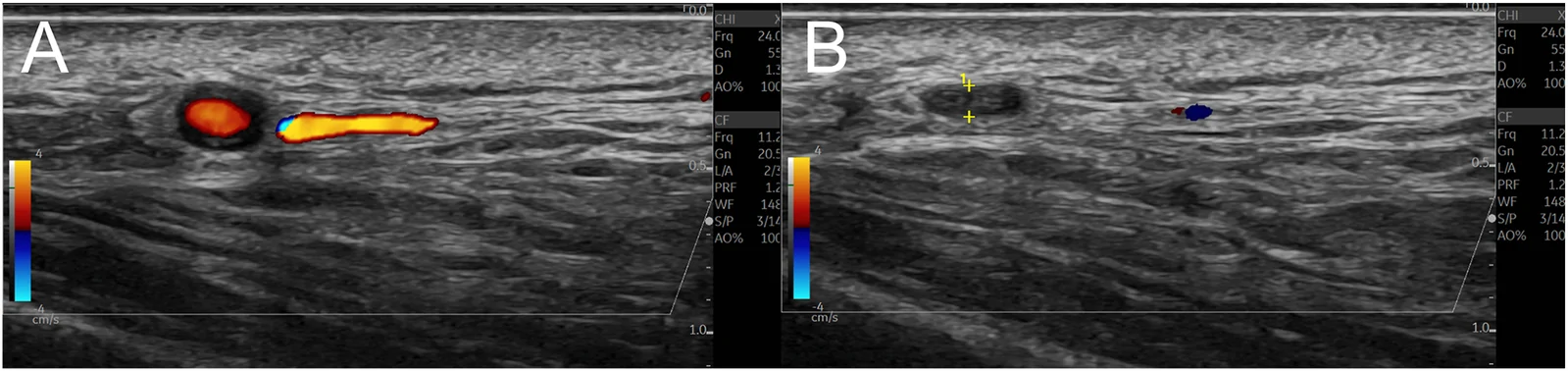
A-B Vascular ultrasound of the right superficial temporal artery. A: Halo sign with color Doppler. B: Compression sign without Doppler with a combined artery wall thickness of 0.9 mm. (For interpretation of the references to color in this figure legend, the reader is referred to the Web version of this article.)

Collectively, the findings of a choroidal perfusion deficit, RAPD, absent optic disc edema, and concurrent GCA were consistent with a diagnosis of A-PION. Dual antiplatelet therapy was discontinued, and the patient followed the local guidelines for corticosteroid tapering in GCA with accompanying vision loss. Throughout 13 months of follow-up to date, CRP has remained below 4 mg/L on monthly samples while the patient has remained on corticosteroid therapy.

At 9 month follow-up visual acuity of the right eye was light perception, and the optic disc was pale and atrophic with a global pRNFL thickness of 34 μm (from 121 μm at presentation). Macular OCT showed pronounced thinning of the inner retinal layers as well as more localized areas of thinning of the inner nuclear layer with an underlying upwards expansion of the outer nuclear layer, characteristic of retinal ischemic perivascular lesions (RIPLs). This demonstrated that the initial retinal ischemic lesions (PAMM) resulted in chronic atrophy of the affected cells (Fig. 3A–B).

**Fig. 3:**
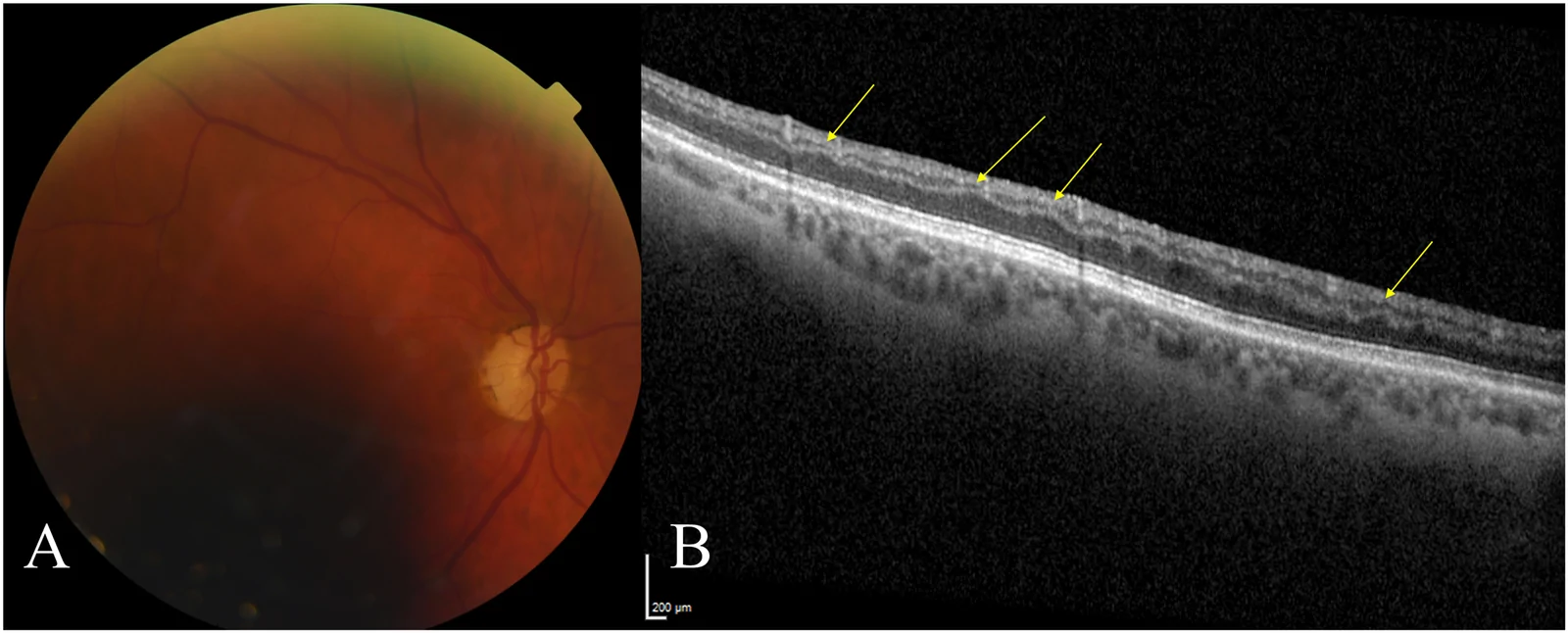
A-B The affected right eye 9 months after the ischemic event. A: Fundus photograph with a pale optic disc. B: Macular optical coherence tomography showing atrophy of inner retinal layers and multiple retinal ischemic perivascular lesions (RIPLs) (arrows).

## 3 Discussion

In this report, we describe the case of a 67-year-old man with sudden monocular vision loss due to A-PION, where PAMM constituted the main finding at initial evaluation. After conducting a literature review on May 1, 2026, utilizing PubMed, Google Scholar, and Embase with the search phrase (('paracentral acute middle maculopathy' or 'PAMM') and ('posterior ischemic optic neuropathy' or 'PION' or 'arteritic PION' or 'A-PION')), we did not find any prior reports of PAMM in the context of A-PION. A-PION is much less common than A-AION and is caused by occlusion of the orbital arteries that supply the posterior (retrobulbar) part of the optic nerve through the pial vascular plexus (predominantly collateral branches from the ophthalmic artery and the posterior ciliary arteries).^9^ The finding of PAMM in a patient with A-PION is consistent with several recent studies reporting an association between PAMM and A-AION or GCA without other ocular manifestations.^3^, ^4^, ^10^, ^11^, ^12^

PAMM is believed to represent intracellular edema in the middle retinal layers, arising from acute ischemia due to hypoperfusion of the deep capillary plexus (DCP). Following the acute phase, PAMM is further characterized by chronic atrophy of the affected cells, manifesting as RIPLs on OCT.^7^ The current understanding of the association between GCA and PAMM is that PAMM reflects hypoperfusion in the central retinal artery (CRA) caused by an incomplete occlusion of the CRA itself or of the ophthalmic artery, secondary to inflammatory remodeling and thickening of the artery wall.^10^, ^11^ As the retinal blood flow predominantly occurs in a serial direction from the superficial capillary plexus (SCP) towards the DCP, the middle retinal layers supplied by the DCP are most vulnerable in the case of global reduction in retinal flow.^13^ However, CWSs resulting from SCP hypoperfusion are also commonly found among GCA patients, often concurrently with PAMM as in the current case.^10^, ^11^ While CWSs have been associated with many other ocular and systemic conditions, including diabetes mellitus and arterial hypertension,^14^ PAMM appears to be a much more specific retinal finding of underlying GCA amongst patients with (anterior) ION.^3^, ^4^

In the present case, ICG angiography further demonstrated a choroidal perfusion deficit, which was important in guiding towards the diagnosis of PION. As PAMM and IONs result from lesions to different orbital arteries, co-occurrence of the two findings should raise a high suspicion of an underlying systemic cause, typically GCA.

The possible association between PAMM and A-PION is a novel finding, but, perhaps more importantly, this case underscores the clinical utility of macular OCT in the initial assessment of patients above 50 years presenting with sudden vision loss. At our center, patients with AION and PION are routinely screened for GCA based on symptoms, age, clinical findings, and inflammatory markers and are referred for further evaluation only on reasonable suspicion. In the present case, inflammatory markers were normal and systemic symptoms absent, and GCA evaluation would therefore not have been prompted by these criteria alone. PAMM may occasionally be the only indication of GCA at presentation, and incorporation of macular OCT into the initial workup of patients with AION and PION may thus improve GCA screening. Sequential involvement of the fellow eye occurs in approximately one-third of patients with visual loss from GCA.^15^ Early detection and treatment is therefore paramount for preserving vision.

The case also demonstrates the utility of ICG angiography in the initial evaluation of patients with monocular vision loss, as it may identify choroidal ischemia, a cardinal sign of underlying GCA that cannot be assessed with structural OCT.^16^

Another important learning point is that reliance on a single diagnostic modality for the assessment of GCA may be insufficient. Regardless of the local choice of first-line diagnostic tool for GCA, a single negative diagnostic test may not effectively rule out GCA when clinical suspicion is high, as sensitivity of each test ranges around 68 – 82%.^8^, ^17^ Therefore, a multimodal approach may be indicated in such cases.

## 4 Conclusion

This case demonstrates that PAMM on macular OCT may occasionally be the only indication of GCA in a patient presenting with sudden vision loss. There is increasing evidence to support that macular OCT should be routinely performed in patients above 50 years presenting with acute vision loss, and that the finding of PAMM should prompt urgent and thorough investigation for GCA and consideration of initiating high-dose corticosteroid therapy, even in the absence of classic diagnostic features.

## CRediT authorship contribution statement

**Andreas Worm Bendtsen:** Writing – original draft, Visualization, Methodology, Investigation, Formal analysis. **Michael Stormly Hansen:** Writing – review & editing, Methodology, Formal analysis, Conceptualization. **Bjarke Askaa:** Writing – review & editing, Methodology, Formal analysis, Data curation. **Steffen Hamann:** Writing – review & editing, Validation, Supervision, Methodology, Conceptualization.

## Ethics declaration

Written informed consent to take part in the study and to publish the article has been obtained from all participants or their legal representatives. The privacy rights of participants have been observed.

This study was performed in compliance with relevant laws, regulatory frameworks and guidelines where the research took place. Ethics committee approval was not required under relevant laws and institutional guidelines.

This research follows the CARE guidelines and the CARE checklist.

## Authorship

All authors attest that they meet the current ICMJE criteria for Authorship.

## Declaration of generative AI and AI-assisted technologies in the manuscript preparation process

Nothing to disclose.

## Funding

No funding or grant support.

## Declaration of competing interest

The authors declare that they have no known competing financial interests or personal relationships that could have appeared to influence the work reported in this paper.
